# Factors associated with lifetime HIV testing among women in four Southeast Asian countries: Evidence from the demographic and health surveys

**DOI:** 10.1177/09564624231162417

**Published:** 2023-03-15

**Authors:** Soe Ohnmar Khin, San Hone, Chunqing Lin, W Scott Comulada, Roger Detels, Sung-Jae Lee

**Affiliations:** 1 Department of Epidemiology, 8783 UCLA School of Public Health, Los Angeles, CA, USA; 2 Maternal and Reproductive Health Division, Department of Public Health, Ministry of Health, Nay Pyi Taw, Myanmar; 3 Department of Psychiatry and Biobehavioral Sciences, 12222 David Geffen School of Medicine at UCLA, Los Angeles, CA, USA; 4 Department of Psychiatry and Biobehavioral Sciences, Department of Health Policy and Management, 8783 UCLA, Los Angeles, CA, USA

**Keywords:** HIV testing, women, prevention of mother-to-child transmission of HIV, Southeast Asian countries, demographic and health surveys

## Abstract

**Background:**

Southeast Asian countries have been trying to increase HIV testing coverage of women since awareness of HIV status is essential to eliminate mother-to-child transmission of HIV. This study determined factors related to lifetime HIV testing uptake among women aged 15–49 years in four Southeast Asian countries: Myanmar, Cambodia, the Philippines and Timor-Leste.

**Methods:**

This study used cross-sectional data from the 2015–16 Myanmar Demographic and Health Survey (DHS), the 2014 Cambodia DHS, the 2017 Philippines National DHS and the 2016 Timor-Leste DHS. We conducted multivariable logistic regression analyses to identify factors associated with lifetime HIV testing among women aged 15–49 years who completed the surveys in each country and ran a fixed effects logistic regression model using pooled data.

**Results:**

The proportions of lifetime HIV testing uptake among women aged 15–49 years were 42.1% in Cambodia, 19.5% in Myanmar, 4.6% in the Philippines, and 3.7% in Timor-Leste. Marital status, age, education, and wealth were significantly associated with lifetime HIV testing uptake among women in all four countries. Other factors (e.g., comprehensive knowledge of HIV, rural/urban residence, positive attitudes towards negotiation for safer sex) were also significant determinants of HIV testing uptake among women in some of these countries.

**Conclusions:**

A multi-sectoral collaboration of related sectors and organizations is necessary to increase access to HIV testing and HIV knowledge of women to overcome the barriers to HIV testing. It is critical to make HIV testing services available and accessible to women, especially in rural areas.

## Introduction

In 2015, approximately 1 in 10 people living with HIV (PLHIV) globally were from the Southeast Asia region.^1^ At the beginning of the epidemic, HIV transmission occurred predominantly among people who inject drugs. However, heterosexual transmission of HIV among people with multiple sexual partners has been increasing in the region since 1989.^2^ In 2015, nearly 39% of an estimated 3.5 million PLHIV in the region were women and girls.^1^ Women in the region are especially at risk for HIV due to gender inequalities, violence against women, and their lower economic, social and legal status.^3^ Furthermore, there has been an increasing number of new HIV infections among the general female population.^3^

Early antiretroviral therapy (ART) and subsequent viral suppression significantly reduce transmission of HIV infection to sexual partners.^4^ Receiving prompt ART depends on early testing for HIV. Women’s awareness of their HIV status is a key step to eliminate vertical transmission of HIV.

Prior studies have found that certain socioeconomic and demographic characteristics – age,^5,6^ marital status,^6–8^ educational levels,^6–8^ occupation,^7^ wealth^5–8^ and place of residence^5,8^ are significantly associated with HIV testing uptake. Moreover, research has demonstrated that comprehensive knowledge of HIV is associated with higher HIV testing uptake,^5,7,9^ while stigma and discrimination of HIV are associated with lower HIV testing uptake.^5^ Additionally, Thapa et al.^10^ found that women with positive attitudes about safer sex negotiation had higher odds of being tested for HIV.

There are few published studies investigating HIV testing uptake among women in Southeast Asian countries. Since the Southeast Asia region has an increasing number of new HIV infections among women at low-risk for HIV,^3^ information regarding factors that hinder or facilitate HIV testing uptake among women is needed to inform policymakers and HIV program implementers. Therefore, this study aimed to assess factors associated with lifetime HIV testing uptake among adult women in four countries – Myanmar, Cambodia, the Philippines and Timor-Leste.

## Methods

This study used the Demographic and Health Surveys (DHS) datasets from four Southeast Asian countries, collected in slightly varying time periods. For Myanmar, the 2015-16 Myanmar DHS (MDHS), for Cambodia, the 2014 Cambodia DHS (CDHS), for the Philippines, the 2017 National DHS (NDHS) and for Timor-Leste, the 2016 Timor-Leste DHS (TLDHS) were used.^11–14^ These datasets are publicly available from the DHS Program upon request.

In 2018, the estimated number of women aged 15 and above living with HIV was 87,000 in Myanmar, 37,000 in Cambodia, 4600 in the Philippines, and 394 in Timor-Leste.^15,16^ UNAIDS estimated that in 2020 in Cambodia 82% of adult females (15+) living with HIV knew their status. ^17^ This percentage was 65% in the Philippines and 92% in Timor-Leste. No data were available for Myanmar women.^17^ These figures highlight HIV testing gaps which leave untreated PLHIV with high viral loads and lead to the further spread of HIV.

The DHS surveys were cross-sectional national surveys using a stratified two-staged sampling design. In the first stage, the surveys selected primary sampling units (PSUs), generally small geographical units within stratified administrative regions, and sampled households in the second stage. All women aged 15–49 years who were either permanent residents of the selected households or visitors who stayed in the household the night before the survey were eligible to respond to the Women’s Questionnaire section of the surveys.^11–14^

All four DHS surveys were interviewer-administered. Interviewers were trained to ensure privacy and check the presence of others before asking sensitive questions. The DHS response rates of eligible women in the four countries were high: 95.8% in Myanmar, 97.6% in Cambodia, 97.6% in the Philippines and 97% in Timor-Leste.^11–14^ We included all women who completed the HIV/AIDS-related questions of the surveys in the four countries: 12,885 in the MDHS dataset, 17,578 in the CDHS dataset, 25,074 in the NDHS dataset, and 4305 in the TLDHS dataset.

The outcome of interest was lifetime HIV testing uptake – “ever been tested for HIV” (yes/no). Potential predictors were socioeconomic and demographic characteristics (age in years, place of residence, current marital status, education level, occupation and wealth index). We included two variables reflecting women’s sense of empowerment. (1) Disagreement that wife-beating is justified for any reason. “Disagreement” means that a woman thinks a husband is not justified in hitting his wife for any reason. Reasons included going out without telling the husband, arguing with him, refusing to have sexual intercourse, neglecting children or burning food. (2) Attitudes towards negotiation for safer sexual relation with husbands. Other potential predictors were exposure to mass media, comprehensive knowledge about HIV and discriminatory attitudes towards PLHIV. Comprehensive knowledge of HIV was defined as knowledge of five facts: (1) consistent use of condoms during sexual intercourse reduces HIV transmission; (2) having just one uninfected faithful partner can reduce the chances of getting HIV; (3) a healthy-looking person can have HIV; and (4/5) reject two common local misconceptions about transmission or prevention of HIV.^18^ The two local misconceptions included in this study were (1) a person can get HIV from mosquito bites and (2) a person can get HIV by sharing food with a person who has AIDS.

We analyzed the data using STATA software (version Stata/SE 15.1). We used ‘svyset’ and ‘svy’ commands and applied sampling weights provided in each country’s dataset to get nationally representative estimates. We conducted separate data analyses for each country and for the pooled sample. Before pooling the four datasets, we de-normalized the weights and gave an equal weight to each country’s survey to avoid over-representation by the countries with larger populations.^19^

Proportions of lifetime HIV testing were compared using Pearson chi-square test corrected for the survey design. Based on the literature and bivariate analyses using weighted Pearson chi-square tests, we selected the predictors that had a bivariate association with lifetime HIV testing at a *p*-value of <0.20 (Table 1). These predictors were included in the multivariable logistic regression models. We included “country” as a covariate in the multivariable logistic regression model for the pooled sample. Multicollinearity of independent variables in the survey data was assessed by calculating tolerance (1-*R*^2^) and VIF.^20^ Model specification was assessed using the “linktest” command.^21^ Model fitting was examined using goodness-of-fit tests for complex survey data.^22^

**Table 1.** Sociodemographic and economic characteristics, HIV related knowledge, attitudes, and sense of empowerment of women aged 15–49 years in the four Southeast Asian countries.

Variables	Myanmar (*n* = 12,885)		Cambodia (*n* = 17,578)		The Philippines (*n* = 25,074)		Timor-Leste (*n* = 4305)	
	No.^a^	%^a^	No.^a^	%^a^	No.^a^	%^a^	No.^a^	%^a^
Marital status								
Never in union	4278	33.2	4428	25.2	8971	35.8	1567	36.4
Currently in union	7759	60.2	11,898	67.7	15,016	59.9	2628	61.0
Formerly in union	848	6.6	1252	7.1	1086	4.3	110	2.5
	*p* < 0.001		*p* < 0.001		*p* < 0.001		*p* < 0.001	
Age in years								
15–19	1810	14.0	2893	16.5	4897	19.5	984	22.8
20–24	1867	14.5	3017	17.2	4175	16.7	782	18.2
25–29	1867	14.5	2836	16.1	3717	14.8	692	16.1
30–39	3990	31.0	4886	27.8	6603	26.3	982	22.8
40–49	3351	26.0	3947	22.5	5682	22.7	866	20.1
	*p* < 0.001		*p* < 0.001		*p* < 0.001		*p* < 0.001	
Highest educational level^b^								
No education or primary	6910	53.6	10,531	59.9	3445	13.7	1629	37.8
Secondary	4646	36.1	6237	35.5	12,491	49.8	2194	51.0
Higher	1325	10.3	810	4.6	9137	36.4	481	11.2
	*p* < 0.001		*p* < 0.001		*p* < 0.001		*p* < 0.001	
Occupation^b^								
Not working	3518	27.4	3729	21.3	11,553	46.2	2735	63.6
Agricultural	1846	14.4	6115	35.0	1390	5.6	477	11.1
Manual	4307	33.5	3971	22.7	5888	23.5	438	10.2
Non-manual	2450	19.1	2891	16.5	2484	9.9	487	11.3
Professional	729	5.7	782	4.5	3705	14.8	165	3.8
	*p* < 0.001		*p* < 0.001		*p* < 0.001		*p* < 0.001	
Wealth index								
Poorest	2274	17.7	3143	17.9	4209	16.8	692	16.1
Poorer	2408	18.7	3314	18.9	4629	18.5	841	19.5
Middle	2633	20.4	3381	19.2	4918	19.6	836	19.4
Richer	2702	21.0	3612	20.6	5527	22.0	941	21.8
Richest	2868	22.3	4128	23.5	5791	23.1	995	23.1
	*p* < 0.001		*p* < 0.001		*p* < 0.001		*p* < 0.001	
Place of residence								
Urban	3768	29.2	3251	18.5	12,252	48.9	1427	33.1
Rural	9117	70.8	14,327	81.5	12,822	51.1	2878	66.9
	*p* < 0.001		*p* < 0.001		*p* < 0.001		*p* < 0.001	
Exposure to any mass media at least once a week								
No	4097	31.8	5520	31.4	3876	15.5	2480	57.6
Yes	8788	68.2	12,058	68.6	21,198	84.5	1824	42.4
	*p* < 0.001		*p* < 0.001		*p* = 0.020		*p* < 0.001	
Comprehensive knowledge of HIV								
No	10,341	80.3	10,710	60.9	18,913	75.4	3891	90.4
Yes	2544	19.7	6868	39.1	6161	24.6	413	9.6
	*p* < 0.001		*p* < 0.001		*p* = 0.001		*p* < 0.001	
Discriminatory attitudes towards PLHIV^c^								
No	5408	42.0	14,127	80.4	10,663	42.5	2950	68.5
Yes	7477	58.0	3451	19.6	14,411	57.5	1355	31.5
	*p* < 0.001		*p* < 0.001		*p* = 0.009		*p* < 0.001	
Disagreement on all of the reasons for wife beating								
No	6592	51.2	8757	49.8	2734	10.9	3207	74.5
Yes	6293	48.8	8821	50.2	22,340	89.1	1098	25.5
	*p* = 0.014		*p* = 0.188		*p* = 0.432		*p* < 0.001	
Attitudes towards negotiation for safer sex^c^								
No	1552	12.0	757	4.3	2285	9.1	2654	61.7
Yes to one question	2593	20.1	4306	24.5	3356	13.4	816	19.0
Yes to both questions	8740	67.8	12,516	71.2	19,433	77.5	834	19.4
	*p* < 0.001		*p* < 0.001		*p* < 0.001		*p* = 0.003	

^a^No. and % are weighted numbers and percentages.

^b^Missing values were excluded. Missing information of highest education level variable was 0.02% in Myanmar. Missing information of occupation variable was 0.23% in Myanmar, 0.59% in Cambodia, 0.22% in the Philippines and 0.09% in Timor-Leste.

^c^Note: Women who have never heard of AIDS were assumed to not have discriminatory attitudes towards PLHIV. The two questions regarding attitudes towards negotiation for safer sex were: (1) If a wife knows her husband has a disease that she can get during sexual intercourse, is she justified in asking that they use a condom when they have sex?; (2) Is a wife justified in refusing to have sex with her husband when she knows he has sex with other women?

Protocols of the Demographic and Health Surveys were approved by the ICF’s Institutional Review Board and an ethical review board in the host country. The publicly available DHS datasets exclude any information that would identify individuals,^23^ and consent waiver was obtained by the ICF’s Institutional Review Board and ethical review board in the host country. Our study was also approved by the Institutional Review Board of the University of California, Los Angeles (Approval Number: IRB#21-001531).

## Results

**Figure 1.**
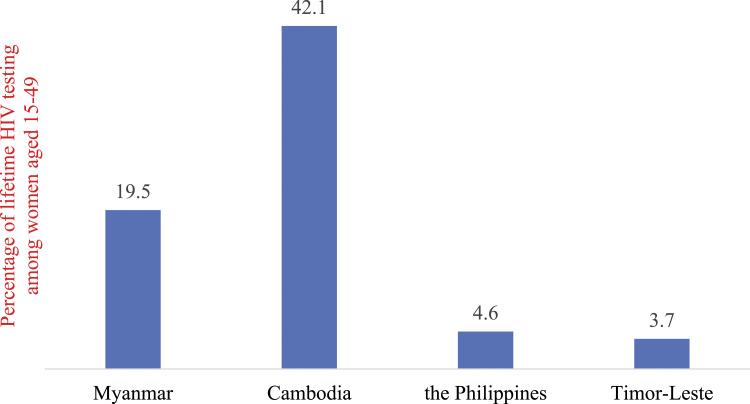
Percentage of lifetime HIV testing among women age15–49 years in four Southeast Asian countries.

The lifetime HIV testing rate among women aged 15–49 years was highest in Cambodia, 42.1% (95% CI: 40.9%, 43.4%) and lowest in Timor-Leste, 3.7% (95% CI: 3.0%, 4.6%) (Figure 1). These percentages were 19.5% (95% CI: 18.3%, 20.7%) in Myanmar and 4.6% (95% CI: 4.0%, 5.2%) in the Philippines. These proportions were significantly different (*p* < 0.001).

Table 1 summarizes sociodemographic and economic characteristics, HIV related knowledge, attitudes, and women’s sense of empowerment in the four countries. Most respondents were currently married, aged 15–24 years (except aged 30–39 in Myanmar), in the richest wealth quintile, from rural areas, and had a lack of comprehensive knowledge of HIV in all four countries. In Myanmar (46.4%), Cambodia (40.1%), the Philippines (86.2%), and Timor-Leste (62.2%) had a secondary or higher level of education. Twenty-seven percent of respondents in Myanmar, 21.3% in Cambodia, 46.2% in the Philippines and 63.6% in Timor-Leste were unemployed. Fifty-eight percent of respondents in Myanmar, 19.6% in Cambodia, 57.5% in the Philippines and 31.5% in Timor-Leste had discriminatory attitudes towards PLHIV. Nearly 90% of respondents from the Philippines (89.1%), almost half of respondents from Myanmar (48.8%) and Cambodia (50.2%) and one-fourth of respondents in Timor-Leste (25.5%) reported disagreement that wife-beating was acceptable. Most respondents in Myanmar (67.8%), Cambodia (71.2%) and the Philippines (77.5%) believed that a wife was justified in (1) refusing to have sexual intercourse with her husband if she knew that he had sex with other women and (2) asking him to use a condom if she knew he had an STI. In Timor-Leste, only 19.4% of respondents had such positive attitudes.

Table 2 shows factors associated with lifetime HIV testing uptake among reproductive aged women in the four countries and the pooled sample. Below we report only those results that were statistically significant for each country and for the pooled sample (See Table 2 for all results). In all four countries and pooled sample, respondents who were currently or formerly married, aged 25–29, and had higher education level had higher odds of being tested for HIV, compared to the respondents who were never married, aged 40–49, and had no education or primary level of education. Respondents who were working in agricultural labor had lower odds of getting tested for HIV than respondents who worked in professional/technical/managerial jobs in the four countries and the pooled sample (aOR 0.42 in Myanmar, 0.55 in Cambodia, 0.33 in the Philippines, 0.34 in Timor-Leste and 0.45 in the pooled sample, respectively).

**Table 2.** Multivariable logistic regression analyses for lifetime HIV testing uptake among women aged 15–49 years by country and pooled sample.

Variables	Myanmar	Cambodia	The Philippines	Timor-Leste^a^	Pooled sample^a^
	aOR (95% CI^†^)	aOR (95% CI)	aOR (95% CI)	aOR (95% CI)	aOR (95% CI)
Country					
Myanmar	NA	NA	NA	NA	1.0 (ref)
Cambodia	NA	NA	NA	NA	3.50 (3.11, 3.92)^b^
The Philippines	NA	NA	NA	NA	0.11 (0.09, 0.13)^b^
Timor-Leste	NA	NA	NA	NA	0.13 (0.10, 0.17)^b^
Marital status					
Never in union	1.0 (ref)	1.0 (ref)	1.0 (ref)	1.0 (ref)	1.0 (ref)
Currently in union	5.95 (4.82, 7.35)^b^	19.54 (16.03, 23.80)^b^	2.02 (1.30, 3.13)^c^	4.61 (2.08, 10.20)^b^	9.05 (7.79, 10.52)^b^
Formerly in union	3.73 (2.83, 4.92)^b^	14.57 (11.24, 18.88)^b^	2.84 (1.69, 4.75)^b^	5.82 (1.89, 17.88)^c^	6.76 (5.61, 8.16)^b^
Age in years					
15–19	0.53 (0.37, 0.76)^b^	3.00 (2.39, 3.76)^b^	0.56 (0.28, 1.12)	1.13 (0.43, 2.97)	1.36 (1.14, 1.62)^c^
20–24	1.81 (1.44, 2.27)^b^	6.97 (5.88, 8.25)^b^	1.54 (0.97, 2.44)	1.83 (0.86, 3.87)	3.46 (3.04, 3.93)^b^
25–29	2.57 (2.12, 3.12)^b^	7.07 (6.02, 8.29)^b^	2.04 (1.40, 2.98)^b^	2.20 (1.08, 4.47)^d^	4.08 (3.60, 4.62)^b^
30–39	2.24 (1.90, 2.63)^b^	3.43 (3.05, 3.86)^b^	1.67 (1.23, 2.28)^c^	1.38 (0.64, 2.98)	2.69 (2.43, 2.99)^b^
40–49	1.0 (ref)	1.0 (ref)	1.0 (ref)	1.0 (ref)	1.0 (ref)
Highest educational level^e^					
No education or primary	1.0 (ref)	1.0 (ref)	1.0 (ref)	1.0 (ref)	1.0 (ref)
Secondary	1.45 (1.25, 1.69)^b^	1.34 (1.20, 1.50)^b^	1.62 (0.97, 2.69)	2.19 (1.22, 3.93)^c^	1.40 (1.28, 1.52)^b^
Higher	2.09 (1.59, 2.76)^b^	1.39 (1.11, 1.75)^c^	2.48 (1.49, 4.11)^b^	3.81 (1.80, 8.08)^c^	2.23 (1.88, 2.66)^b^
Occupation^e^					
Professional	1.0 (ref)	1.0 (ref)	1.0 (ref)	1.0 (ref)	1.0 (ref)
Agricultural	0.42 (0.29, 0.61)^b^	0.55 (0.43, 0.71)^b^	0.33 (0.18, 0.61)^b^	0.34 (0.11, 1.00)^d^	0.45 (0.36, 0.55)^b^
Manual	0.60 (0.44, 0.84)^c^	0.84 (0.64, 1.10)	0.60 (0.40, 0.91)^d^	0.92 (0.43, 1.97)	0.65 (0.53, 0.80)^b^
Non-manual	0.67 (0.49, 0.91)^c^	0.81 (0.63, 1.04)	0.73 (0.47, 1.13)	0.56 (0.28, 1.12)	0.66 (0.54, 0.81)^b^
Not working	0.81 (0.59, 1.11)	0.90 (0.69, 1.18)	0.43 (0.30, 0.62)^b^	0.47 (0.23, 0.93)^d^	0.66 (0.54, 0.81)^b^
Wealth index					
Poorest	1.0 (ref)	1.0 (ref)	1.0 (ref)	1.0 (ref)	1.0 (ref)
Poorer	1.33 (1.07, 1.67)^d^	0.98 (0.83, 1.16)	1.64 (0.99, 2.73)	0.85 (0.26, 2.74)	1.15 (1.02, 1.30)^d^
Middle	1.48 (1.16, 1.89)^c^	1.08 (0.91, 1.30)	2.01 (1.20, 3.37)^c^	1.21 (0.37, 3.89)	1.33 (1.16, 1.51)^d^
Richer	1.87 (1.46, 2.40)^b^	1.09 (0.90, 1.32)	2.03 (1.16, 3.52)^d^	1.15 (0.34, 3.85)	1.44 (1.25, 1.65)^c^
Richest	2.34 (1.74, 3.13)^b^	1.63 (1.29, 2.06)^b^	3.00 (1.66, 5.44)^c^	1.89 (0.58, 6.21)	2.05 (1.73, 2.43)^b^
Place of residence					
Urban	1.0 (ref)	1.0 (ref)	1.0 (ref)	1.0 (ref)	1.0 (ref)
Rural	0.73 (0.59, 0.89)^c^	0.70 (0.59, 0.84)^b^	0.43 (0.30, 0.62)^b^	0.86 (0.48, 1.55)	0.68 (0.60, 0.77)^b^
Exposure to any mass media at least once a week					
No	1.0 (ref)	1.0 (ref)	1.0 (ref)	1.0 (ref)	1.0 (ref)
Yes	1.01 (0.86, 1.19)	1.23 (1.11, 1.37)^b^	1.02 (0.71, 1.46)	1.40 (0.88, 2.24)	1.12 (1.02, 1.23)^d^
Comprehensive knowledge of HIV					
No	1.0 (ref)	1.0 (ref)	1.0 (ref)	1.0 (ref)	1.0 (ref)
Yes	1.36 (1.18, 1.57)^b^	1.28 (1.16, 1.41)^b^	0.97 (0.73, 1.29)	1.92 (1.14, 3.22)^d^	1.33 (1.22, 1.45)^b^
Discriminatory attitude towards PLHIV^f^					
No	1.0 (ref)	1.0 (ref)	1.0 (ref)	1.0 (ref)	1.0 (ref)
Yes	0.93 (0.83, 1.05)	0.67 (0.58, 0.76)^b^	0.82 (0.63, 1.08)	2.86 (1.61, 5.08)^b^	0.93 (0.85, 1.02)
Disagreement over all the reasons for wife beating					
No	1.0 (ref)	1.0 (ref)	-^g^	1.0 (ref)	1.0 (ref)
Yes	0.93 (0.83, 1.05)	0.96 (0.88, 1.06)		0.31 (0.17, 0.55)^b^	0.90 (0.84, 0.97)^c^
Attitude towards negotiation for safer sex^f^					
No	1.0 (ref)	1.0 (ref)	1.0 (ref)	1.0 (ref)	1.0 (ref)
Yes to one question	1.54 (1.17, 2.02)^c^	2.31 (1.63, 3.26)^b^	1.30 (0.63, 2.68)	0.60 (0.31, 1.17)	1.35 (1.10, 1.66)^c^
Yes to both questions	2.03 (1.53, 2.70)^b^	2.30 (1.64, 3.23)^b^	1.59 (0.85, 2.98)	0.91 (0.54, 1.55)	1.59 (1.30, 1.95)^b^

NA, not applicable; aOR, adjusted odds ratio; CI, confidence interval; ref, reference.

^a^The multivariable logistic regression models of Timor-Leste and pooled sample had *p* value <0.05 in the goodness of fit test.

^b^*p* value <0.001.

^c^*p* value <0.01.

^d^*p* value <0.05.

^e^Missing values were excluded from the multivariable analyses. Percentage of missing observations of “^a^ variables” ranged from 0.01% to 0.59%.

^f^Note: Women who have never heard of AIDS were assumed to not have discriminatory attitudes towards PLHIV. The two questions regarding attitudes towards negotiation for safer sex were: (1) If a wife knows her husband has a disease that she can get during sexual intercourse, is she justified in asking that they use a condom when they have sex?; (2) Is a wife justified in refusing to have sex with her husband when she knows he has sex with other women?

^g^The variable was not included in the multivariable logistic regression model as *p* value was ≥0.2 in the bivariate analysis.

In Myanmar, Cambodia, the Philippines, and the pooled sample, the richest wealth quintile was significantly associated with higher odds of lifetime HIV testing compared to the poorest quintile, while living in rural areas was significantly associated with lower odds of lifetime HIV testing compared to living in urban areas. In Myanmar, Cambodia, Timor-Leste and the pooled sample, respondents with comprehensive knowledge of HIV had higher odds of being tested for HIV than those who did not have this knowledge (aOR 1.36, 1.28, 1.92 and 1.33 respectively). In Timor-Leste and the pooled sample, women who disagreed with wife beating had lower odds of getting tested for HIV (aOR 0.31 and 0.90). In Myanmar, Cambodia, and the pooled sample, higher odds of lifetime HIV testing was found among respondents with positive attitudes towards negotiation for safer sexual relations with husbands (aOR 2.03, 2.30 and 1.59 respectively).

## Discussion

Many of our potential predictors were found to be significant in most of the four countries and the pooled sample. Overall, this study identified gaps in HIV testing among women who were never married, had low education levels, were poor, lived in rural areas, and lacked comprehensive knowledge of HIV.

The finding that in all four countries, ever-married women were more likely to get tested for HIV is consistent with previous studies.^6,7^ Ever-married women have more chances of being tested for HIV through services aimed at prevention of mother-to-child transmission (PMTCT) of HIV. Consistent with previous studies,^6–8^ we found that HIV testing uptake of women increased as their education level increased. Such an association is possibly due to the impact of sexual and reproductive health education programs offered at higher levels of education. These results highlight the need to expand health education programs about HIV/AIDS and reproductive health to lower levels of education.

The respondents in the middle and above wealth quintiles were more likely to get tested than those in the poorest, in agreement with the results from previous studies.^6–8^ Women with more assets have greater access to health care services and can pay for additional testing-associated costs beyond the free tests. Therefore, an important recommendation is to provide mechanisms to help poor people cover all out-of-pocket costs for getting an HIV test.

Consistent with the study by Gazimbi and Magadi in Zimbabwe,^5^ our results showed that in Myanmar, Cambodia and the Philippines, respondents from rural areas were less likely to get tested than those from urban areas. In developing countries generally there are fewer and under-staffed health facilities in rural areas. Recruiting local community health workers, providing mobile testing and self-testing for HIV would make the services more available to rural residents.

Comprehensive knowledge of HIV was a significant predictor for lifetime HIV testing uptake in Myanmar, Cambodia and Timor-Leste. Our findings are consistent with those from Ethiopia,^7^ Zimbabwe,^5^ Kenya and Mozambique.^9^ Evidence of a positive association between comprehensive knowledge of HIV and HIV testing uptake stresses the importance of continued and effective dissemination of health messages about HIV/AIDS, HIV testing services and the benefits of getting tested.

Our results showed that in Myanmar and Cambodia the likelihood of HIV testing uptake was high among women who had positive attitudes towards negotiation for safer sexual relations with husbands. A study in Nepal also highlighted a positive association between attitudes towards safer sex negotiation and HIV testing uptake.^10^ Women’s sense of empowerment regarding sexual and reproductive health decision making is necessary to increase their HIV testing uptake.

Besides the similarity in results among these countries, there were also important differences regarding the percentages of lifetime HIV testing uptake of women: 19.5% in Myanmar, 42.1% in Cambodia, 4.6% in the Philippines and 3.7% in Timor-Leste.

In 2013, Myanmar initiated the decentralization of HIV testing services (HTS), that is, shifted the testing process from laboratory-based testing to rapid HIV tests by basic health care workers. As a result, the numbers of people being tested and receiving HTS significantly increased from 2014 to 2015.^24^ Similarly, the percentage of HIV testing uptake increased among women who attended antenatal care (ANC) and received pre-test counselling from 51% in 2013 to 87% in 2015.^24^ However, in the general population based on our results, only 2 in 10 Myanmar women aged 15–49 had ever been tested for HIV. Therefore, this study underscores the need for HTS to target non-pregnant women of reproductive age, especially those who are partners of key populations.

In Cambodia, many factors could explain higher HIV testing rates among women compared to the other three countries. Cambodia was one of the first Southeast Asia countries to experience the HIV epidemic and initiate a multisectoral and effective response. Implementation of the “Linked Response” approach greatly increased the proportion of ANC attendees tested for HIV.^25^ For pregnant women who never visit ANC facilities, outreach ANC provides HIV testing at the community level.^26^ Furthermore, HIV testing is free and HIV services are well funded.^27,28^

In the Philippines, predominantly male-to-male sexual transmission over the last decades,^29^ modest awareness of HIV testing among women of reproductive age^14^ and an absence of a free HIV testing policy^28^ could be reasons for the very low HIV testing rates among women aged 15–49 years. Recent significantly rising numbers of HIV infections among adolescent girls and young women through sexual contact suggest that heterosexual transmission is now driving the HIV epidemic in the Philippines.^29^ Therefore, it is extremely critical to take actions to increase HIV testing uptake among young women, especially adolescent girls.

In our study, the percentage of HIV testing uptake among Timorese women aged 15–49 was strikingly low (3.7%). This finding might be explained by the fact that HIV prevalence in Timor-Leste is very low and concentrated among key populations. In Timor-Leste, there is limited access to HIV services.^30^ In 2014, less than half of the districts were covered by the PMTCT program, and only 19.3% of pregnant women had been tested for HIV and received their results.^15^ Furthermore, the proportion of women who have HIV prevention knowledge and who know HIV testing places was very low.^11^

There are limitations of our study. Since the findings were based on cross-sectional survey data, we could not ascertain the temporality of the reported associations. Hence, we cannot make causal interpretations. We used the term “lifetime HIV testing uptake” as our outcome variable but many of our predictors contained time varying responses such as knowledge of HIV, discriminatory attitudes and wealth index. Therefore, “lifetime” interpretation should be considered with this caveat in mind. The surveys covered slightly different years, but all four were conducted between 2014 – 2017: and we do not feel that the study period contributed to significant differences between the four countries. As the disagreement about wife-beating variable was omitted in regression model for the Philippines, comparisons of the Philippines with the other three countries should be interpreted with this omission in mind.

Additionally, results from the pooled sample should be interpreted with caution since it combined data from four countries. The low percentage of HIV testing uptake and the small sample size in Timor-Leste resulted in wide 95% confidence intervals. Another limitation of our research is that we did not study certain factors which also may be responsible for the differences in HIV testing uptake. These factors include the characteristics of the HIV epidemic in each country as well as the availability of HIV prevention resources.

Despite these limitations, our study has several strengths. Our results are based on large, nationally representative samples. The DHS program provided high quality and reliable data: well-designed questionnaires, trained interviewers, and high response rates. The pooled sample also provided stable estimates. The sample design and implementation of the surveys reduced selection bias. Lastly, comparison of indicators between countries was possible due to the use of standardized questionnaires.

## Conclusion

To increase HIV testing uptake among women of reproductive age in these four countries, a multisectoral collaboration of related sectors and organizations is necessary to increase HIV knowledge and increase access to HIV testing, including options for self-testing, and empowering women to make their own decision around safer sex and HIV testing. Our study underscored evidence that high knowledge of HIV increases HIV testing among women. Peer-led or community-led health education about HIV/AIDS will increase HIV testing uptake. It is critical to take action for making HIV testing services available and accessible to women in rural areas. This includes providing options for self-testing and partner testing of HIV and integration of HIV testing services in sexual and reproductive health and rights (SRHR) activities.

Strategies to fill the gaps in HIV testing uptake should be context-specific and depend on the nature of the epidemic, the organizational structure of health service delivery, available resources, collaborations with related stakeholders, and especially importantly, with community participation. A combination of these approaches in these four countries will contribute to their achieving the UNAIDS 95-95-95 targets by 2025 and to ending their AIDS epidemic by 2030.
